# Detailed statistical analysis plan for the short-term versus long-term mentalisation-based therapy for outpatients with subthreshold or diagnosed borderline personality disorder randomised clinical trial (MBT-RCT)

**DOI:** 10.1186/s13063-021-05450-y

**Published:** 2021-07-28

**Authors:** Sophie Juul, Sebastian Simonsen, Stig Poulsen, Susanne Lunn, Per Sørensen, Anthony Bateman, Janus Christian Jakobsen

**Affiliations:** 1 Stolpegaard Psychotherapy Centre, Mental Health Services in the Capital Region of Denmark, Stolpegårdsvej 20, 2820 Gentofte, Denmark; 2 Department of Psychology, University of Copenhagen, Copenhagen, Denmark; 3 Copenhagen Trial Unit, Centre for Clinical Intervention Research, Department 7812, Rigshospitalet, Copenhagen University Hospital, Copenhagen, Denmark; 4 Anna Freud Centre, Kantor Centre of Excellence, London, UK; 5 Department of Regional Health Research, The Faculty of Health Sciences, University of Southern Denmark, Odense, Denmark

## Abstract

**Background:**

Psychotherapy for borderline personality disorder is often extensive and resource-intensive. Mentalisation-based therapy is a psychodynamically oriented treatment option for borderline personality disorder, which includes a case formulation, psychoeducation, and group and individual therapy. The evidence on short-term compared with long-term mentalisation-based therapy is currently unknown.

**Methods/design:**

The Short-Term MBT Project (MBT-RCT) is a single-centre, parallel-group, investigator-initiated, randomised clinical superiority trial in which short-term (20 weeks) will be compared with long-term (14 months) mentalisation-based therapy for outpatients with subthreshold or diagnosed borderline personality disorder. Outcome assessors, data managers, the data safety and monitoring committee, statisticians, and decision-makers will be blinded to treatment allocation. Participants will be assessed before randomisation and at 8, 16, and 24 months after randomisation. The primary outcome will be the severity of borderline symptomatology assessed with the Zanarini Rating Scale for Borderline Personality Disorder. Secondary outcomes will be functional impairment (Work and Social Adjustment Scale), quality of life (Short-Form Health Survey 36—mental component), global functioning (Global Assessment of Functioning), and proportion of participants with severe self-harm. In this paper, we present a detailed statistical analysis plan including a comprehensive explanation of the planned statistical analyses, methods to handle missing data, and assessments of the underlying statistical assumptions. Final statistical analyses will be conducted independently by two statisticians following the present plan.

**Discussion:**

We have developed this statistical analysis plan before unblinding of the trial results in line with the Declaration of Helsinki and the International Conference on Harmonization of Good Clinical Practice Guidelines, which should increase the validity of the MBT-RCT trial by mitigation of analysis bias.

**Trial registration:**

ClinicalTrials.gov NCT03677037. Registered on 19 September 2018

**Supplementary Information:**

The online version contains supplementary material available at 10.1186/s13063-021-05450-y.

## Background

Mentalisation-based therapy (MBT) is a long-term, psychodynamically oriented psychotherapy developed specifically to treat patients with borderline personality disorder [1]. Even though more randomised clinical trials at low risk of bias are still needed, long-term MBT is considered one of the most evidence-based interventions currently available for patients with borderline personality disorder [2]. However, the optimal duration of MBT for borderline personality disorder is currently unclear.

The Short-Term MBT Project (MBT-RCT) is a single-centre, parallel-group, investigator-initiated, randomised clinical superiority trial with the objectives to assess the beneficial and harmful effects of short-term (20 weeks) compared with long-term (14 months) MBT for outpatients with subthreshold or diagnosed borderline personality disorder [3]. The Helsinki Declaration [4] and the International Conference on Harmonization of Good Clinical Practice (ICH-GCP) [5] Guidelines recommend that clinical trials should be analysed according to a pre-specified plan to prevent selective outcome reporting bias and data-driven analysis results [6–8].

In this publication, we describe the pre-planned statistical analyses of the primary and secondary outcomes in the MBT-RCT trial. The main publication of the trial results will adhere to this statistical analysis plan as approved by the steering group.

## Methods

The design of the MBT-RCT trial has been described in detail previously [3]. The trial population will be adults (18 years of age or older) with subthreshold or diagnosed borderline personality disorder assessed with the Structural Clinical Interview for DSM-5 Personality Disorders (SCID-5-PD). Participants will be eligible for enrolment if they meet all of the following inclusion criteria and none of the exclusion criteria as presented in Table 1.

**Table 1:** Inclusion and exclusion criteria

	General criteria of the outpatient clinic	Criteria exclusive to the trial
**Inclusion criteria**	• Aged 18–60 • Personality disorder(s) considered to be the primary diagnosis	• A minimum of four confirmed DSM-5 diagnostic criteria for borderline personality disorder • Written informed consent
**Exclusion criteria**	• Learning disability (IQ < 75) • A full diagnosis of schizotypal personality disorder or antisocial personality disorder • Presence of a comorbid psychiatric disorder that requires specialist treatment • Current (past 2 months) substance dependence including alcohol • Concurrent psychotherapeutic treatment outside the clinic	• Unable to understand Danish • Lack of informed consent

The MBT-RCT trial is registered at ClinicalTrials.gov (identifier: NCT03677037), is carried out in compliance with the Declaration of Helsinki [4], and is approved by the Danish Data Protection Agency (approval number: 6553) and by the Regional Research Ethics Committee of the Capital Region of Denmark (approval number: H-18023136).

### Randomisation and blinding

Copenhagen Trial Unit, a Danish centre for clinical intervention research, will be responsible for the central randomisation. Randomisation will be performed with a 1:1 allocation according to a computer-generated allocation sequence with permuted blocks of various sizes. The allocation sequence will be concealed from all trial investigators. The randomisation is stratified by (1) sex and (2) high/low baseline scores on the primary outcome measure, the Zanarini Rating Scale for Borderline Personality Disorder (ZAN-BPD) [9].

Outcome assessors, data managers, statisticians, the data safety and monitoring committee, and decision-makers will be blinded to treatment allocation [10]. Trial participants and therapists will not be blind to the treatment allocation. This is due to the difficulties of implementing an efficient blinding procedure in trials assessing psychological interventions [10].

### Trial interventions

#### Experimental intervention

The short-term MBT programme is organised as a 20-week programme consisting of 20 weeks of group therapy in closed groups commencing with five sessions of psychoeducative introduction to MBT [11] followed by 15 sessions of group MBT group therapy accompanied by conjoined individual sessions every second week. All participants are furthermore invited to two psychoeducative meetings with other participants and their relatives. The participants will be treated by two group therapists, one of them also being the individual therapist. Both group and individual therapies are manualised by Bateman and Fonagy [1].

#### Control intervention

Long-term MBT is organised as a 14-month programme and has been implemented at the clinic for the past 10 years. All participants randomised to long-term MBT will initially enter a 6-week psychoeducative introduction to MBT [11]. New psychoeducative MBT groups commence every time new participants are recruited and randomised to long-term MBT. When the psychoeducative group finishes, participants will be allocated to one of eight slow-open MBT treatment groups. Treatment is then organised as 12 months of weekly group therapy sessions combined with individual therapy every second week. All participants are furthermore invited to two psychoeducative meetings with other participants and their relatives. The participants will be treated by two group therapists and a third individual therapist. Both group and individual therapies are manualised by Bateman and Fonagy [1].

### Baseline characteristics

The baseline characteristics will be assessed from inclusion in the trial. A mock table of the complete pre-defined baseline table can be found in Supplementary Material 1. The baseline characteristics will be as follows:

1. Demographic characteristics:
  - Age
  - Sex
  - Civil status
  - Living situation (alone/with others)
  - Education level
  - Employment status
2. Clinical characteristics:
  - Psychiatric comorbidity, e.g. a diagnosis of an anxiety disorder, major depressive disorder, post-traumatic stress disorder (reported if the frequency is above or equal to 10% in any of the intervention groups)
  - Proportion of participants with subthreshold borderline personality disorder
  - Mean number of borderline personality disorder diagnostic criteria
  - Personality disorder comorbidity (reported if the frequency is above or equal to 10% in any of the intervention groups)
  - Proportion of participants with one or more suicide attempts 8 months prior to randomisation
  - Proportion of participants with severe self-harm incidents defined as deliberate acts of self-harm resulting in visible tissue damage 8 months prior to randomisation
  - Proportion of participants on psychopharmacological medication (e.g. antidepressants, antipsychotics) at baseline
3. Mean (SD) number of days from randomisation to assessment time point for all time points (8, 16, and 24 months post-randomisation)

### Outcomes

The outcomes were predefined as primary, secondary, and exploratory [3]. This publication describes the statistical analysis plan of the primary and secondary outcomes only.

#### Primary outcome

- Severity of borderline symptomatology assessed with the Zanarini Rating Scale for Borderline Personality Disorder [9]

#### Secondary outcomes

- Functional impairment (assessed with the Work and Social Adjustment Scale (WSAS) [12])
- Quality of life (assessed with the Short-Form Health Survey (SF-36) mental component) [13]
- Global functioning (assessed with the Global Assessment of Functioning scale (GAF)) [14]
- Severe self-harm (defined as the proportion of participants with one or more deliberate acts of self-harm resulting in visible tissue damage)

#### Exploratory outcomes

- Symptom distress (assessed with the Symptom Checklist 90 (SCL-90)) [15]
- Quality of life (assessed with the Short-Form Health Survey (SF-36) physical component [13])

### Assessment time points

All outcomes will be assessed at baseline and at 8, 16, and 24 months after randomisation. Investigator-administered outcomes (severity of borderline symptomatology, severe self-harm, and global functioning) will be assessed by blinded assessors at all time points. We will use the 16-month time point as the primary time point of interest, as it is the time point closest to the end of treatment in the long-term MBT group. In an exploratory analysis, we will consider reporting the results of the comparison between the end of treatment in both groups (i.e. data from the 8 months time point in the short-term group compared with data from the 16 months time point in the long-term group). Data from the 24-month time point, as well as results of the exploratory outcomes, will be analysed using the same principles as described in this statistical analysis plan and published in a separate publication.

### Safety

We will report the proportion of participants with one or more serious adverse events in both groups. We will use the International Conference on Harmonisation of technical requirements for registration of pharmaceuticals for human use—Good Clinical Practice (ICH-GCP) definition of a serious adverse event, which is any untoward medical occurrence that resulted in death, was life-threatening, required hospitalisation or prolonging of existing hospitalisation, and resulted in persistent or significant disability or jeopardised the participant [5]. Two investigators will independently go through the participants’ medical journals and assess possible serious adverse events at the 16 and 24 months time point of assessment according to the ICH-GCP definition.

### Sample size and power estimations

The sample size estimation was based on the primary outcome, and our primary conclusions will be based on the results of the primary outcome. The outcomes in our outcome hierarchy were ranked according to clinical relevance, and we estimated the power of each non-primary outcome to ensure that we had sufficient power to confirm or reject minimally important intervention effects [16].

#### Sample size estimation

The sample size was determined by the predicted change in the primary outcome measure, ZAN-BPD. We considered a 3.5-point superiority margin to be the minimal important difference. Consistent with previous trials that have used ZAN-BPD as an outcome measure for a group of participants similar to ours [17, 18] we expect a standard deviation of 8. With power set at 80% and alpha set at 5% two-tailed, a sample size of 83 participants will be needed in each intervention group, corresponding to a total of 166 participants.

We did not adjust the sample size according to missing data. We plan to have as close to 0% missing data as possible. However, if missing data occur, we will use multiple imputations which will limit the loss of power (please see the ‘Handling of missing data’ section).

#### Power estimation for secondary outcomes

For the secondary outcomes, we have performed power calculations as presented in Tables 2 and 3 [16].

**Table 2:** Power estimations for the secondary continuous outcomes

Outcome	Minimal clinically important difference	Expected standard deviation	Alpha	Sample size	Power	Reference
	*δ*	*σ*	*α*	*n*	1−*β*	
Functional impairment (WSAS)	4.5	10	5	166	82	Phillips et. al. [19]
Quality of life (SF-36—mental component)	5	11	5	166	83	Rollman et al. [20]
Global functioning (GAF)	4	8.5	5	166	85	Jørgensen et al. [21]

*GAF* Global Assessment of Functioning, *SF-36* Short-Form Health Survey 36, *WSAS* Work and Social Adjustment Scale

**Table 3:** Power estimations of secondary dichotomous outcomes

Outcome	Expected proportion in the control group	Alpha significance level	Power	Reference
Severe self-harm incidents^a^	15%	5	14%	Simonsen et al. [22]; Bateman and Fonagy [23]

^a^Even though the expected power is only 14%, we define severe self-harm incidents as a secondary outcome, because it is considered very important for this population

### General analysis principles

Statistical analyses will be performed in Stata [24]. All analyses will be conducted according to the intention-to-treat principle (ITT). The intention-to-treat population will include all randomised participants, regardless of missing data, lost to follow-up, or adherence to the intervention. Thus, by performing an intention-to-treat analysis, we will assess the effects of being randomised to the interventions. We will consider performing a per-protocol analysis, if the number of participants who prematurely drops out of treatment exceeds 5% of the total trial population. By performing a per-protocol analysis, we will assess the effects of adhering to the intervention, which must be considered hypothesis-generating only.

It is generally recommended that regression analyses should be adjusted for the stratification variables used in the randomisation [25–27]. Thus, all analyses will primarily be adjusted for the stratification variables used in the randomisation (and the baseline value of the outcome of interest when assessing continuous outcomes). We will secondly adjust all analyses for the following adjustment variables: age (18–30/31–60), baseline global functioning as assessed with the GAF score (0–48/49–100), baseline proportion of participants with severe self-harm 8 months prior to randomisation (participants with one or more events/participants with no events), and proportion of participants who had their group therapy temporarily paused due to COVID-19 in March 2020 and January 2021 compared to the proportion of participants who did not have their group therapy temporarily paused.

We will perform the following subgroup analyses (test of interaction):

- Baseline severity of borderline symptomatology (ZAN-BPD scores 0–11/12–36)
- Sex (male/female)
- Age (18–30/31–60)
- Baseline global functioning (GAF scores 0–48/49–100)
- Baseline proportion of participants with severe self-harm incidents 8 months prior to randomisation (participants with one or more events/participants with no events)
- Proportion of participants who had their group therapy temporarily paused due to COVID-19 in March 2020 and January 2021 compared to participants who did not have their group therapy temporarily paused

We will present the results of the subgroup analyses in forest plots.

### Trial profile

The flow of trial participants will be displayed in a Consolidated Standards of Reporting Trials (CONSORT) diagram [28]. The number of screened patients who were assessed for eligibility, and the number included in the primary and secondary analyses, as well as all reasons for exclusions in the primary and secondary analyses, will be reported.

### Statistical analyses

#### Analysis of continuous data

Continuous outcomes will be presented as means and standard deviations for each group together with 95% confidence intervals for the means of the groups and the mean differences between the groups. We will analyse the continuous outcomes using linear regression. All variables will be included as fixed effects.

#### Analysis of dichotomous data

Dichotomous outcomes will be presented as proportions of participants in each group with the event, together with risk ratios with 95% confidence intervals. We will analyse the dichotomous outcomes using logistic regression. All variables will be included as fixed effects. Odds ratios will be transformed to risk ratios estimating marginal effects using the NLCOM command in Stata [24].

### Level of significance

The threshold for significance will be assessed according to a five-step procedure, suggested by Jakobsen and colleagues [29].

The first step will be to calculate and report confidence intervals and *p*-values for the primary and secondary outcomes. All confidence intervals will be 95% and two-sided. We will use a *p*-value of less than 0.05 as the threshold for statistical significance for our primary outcome (see the ‘Sample size estimation’ section) since we plan to report on only one primary outcome. Since our primary conclusions will be based on one outcome result at one time point (16 months post-randomisation), we will limit problems associated with multiple testing due to multiple outcome comparisons [30, 31]. All remaining outcome results and assessment time points will be considered hypothesis-generating only.

The second step will be to calculate and report the Bayes factor [32] for primary and secondary outcomes. The Bayes factor is the ratio between the probability of the results given that the null hypothesis (*H*_0_) is true divided by the probability of the results given that the alternative hypothesis (*H*_A_) is true [32]. Calculating and reporting the Bayes factor will allow us to interpret the results of the primary outcome in relation to former trial results [17, 18].

The third step will be to use Lan-DeMets monitoring boundaries if the trial is stopped before the sample size is reached [33]. This is done to avoid a potential false rejection of the null hypothesis caused by an insufficient sample size [34].

The fourth step regarding adjustment of *p*-values based on multiple testing of the primary outcome is not applicable to our trial. We only have one single primary outcome, primarily assessed at one time point (16 months post-randomisation) [29].

The fifth step is the assessment of the clinical significance. The assessment of the clinical significance of our trial results will be based on the intervention effects we predefined in the sample size and power estimations.

### Interim analyses

We have pre-planned one interim analysis, which will be conducted after half of the trial participants have been assessed at the 8 months post-randomisation time point. The timing and prevalence of any additional interim analyses will be decided exclusively by the members of the data monitoring and safety committee. The role of the data monitoring and safety committee will be to make recommendations to the steering group to either continue, change, hold, or terminate the trial. This recommendation will primarily be based on safety considerations. The data monitoring and safety committee will be provided with the following trial data: number of participants randomised, number of participants per intervention group, baseline ZAN-BPD scores for all participants, ZAN-BPD scores at the 8-month post-randomisation time point for participants in both intervention groups with available data at that time point, proportion of participants with one or more deliberate acts of self-harm at the 8-month post-randomisation time point, and serious adverse events. Based on evaluations of these data, the data monitoring and safety committee will decide whether they want further data from the principal investigator and when next to perform analyses on data.

### Handling of missing data

Missing data will be handled according to the recommendations of Jakobsen and colleagues [35]. In short, if we experience missing data, we will consider to use multiple imputations and use best-worst/worst-best case scenarios to assess the potential impact of the missing data [35].

All randomised participants (the intention-to-treat population) will be included in the primary analysis of all outcomes. If it is not valid to ignore missing data (that is, if the missing data exceeds 5%), we will consider using multiple imputations and use best-worst/worst-best case scenarios to assess the potential impact of the missing data [35]. Best-worst and worst-best case scenarios assess the potential range of impact of the missing data for the trial results [35]. In the ‘best-worst’ case scenario, it is assumed that all participants lost to follow-up in the short-term group have had a beneficial outcome (e.g. had no self-harm incidents), and all those in the long-term group have had a harmful outcome (e.g. had one or more self-harm incidents). Conversely, in the ‘worst-best’ case scenario, it is assumed that all participants who were lost to follow-up in the short-term group have had a harmful outcome and that all those lost to follow-up in the long-term group have had a beneficial outcome [35]. When continuous outcomes are used, a ‘beneficial outcome’ will be defined as the group mean plus two SDs of the group mean (fixed imputation), and a ‘harmful outcome’ will be defined as the group mean minus two SDs of the group mean (fixed imputation) [35]. We do not expect any missing baseline data, as participants will only be randomised once they have a complete baseline dataset.

### Assessments of underlying statistical assumptions

We will assess the underlying statistical assumptions for all statistical analyses [36, 37]. We will test for major interactions between each covariate and the intervention variable for all regression analyses. We will, in turn, include each possible first-order interaction between included covariates and the intervention variable. For each combination, we will test if the interaction term is significant and we will assess the effect size. We will only consider concluding that there is evidence of an interaction if (1) the interaction is statistically significant following the Bonferroni-adjusted thresholds (0.05 divided by the number of possible interactions) and (2) if the interaction shows a clinically significant effect. If we conclude that the interaction is statistically significant, we will consider both presenting a separate analysis for each interaction as well as an overall analysis including the interaction term in the model [36].

#### Assessments of underlying statistical assumptions for linear regression

We will visually inspect quantile-quantile plots of the residuals [38, 39] to assess if the residuals are normally distributed, and we will use residuals plotted against covariates and fitted values [38, 39] to assess for homogeneity of variances. If the plots show deviations from the model assumptions, we will consider transforming the outcome, i.e. by using log transformation or square root and/or use robust standard errors [38, 39].

#### Assessments of underlying statistical assumptions for logistic regression

We will assess if the deviance divided by the degrees of freedom is significantly larger than 1 to assess for relevant overdispersion. If that is the case, we will consider using a maximum likelihood estimate of the dispersion parameter.

### Statistical reports

Two independent statisticians will analyse blinded data on all outcomes with intervention groups concealed as, e.g., ‘A’ and ‘B’. Two independent statistical reports will be delivered to the principal investigator (SJ) and will be shared with the steering group. If there are discrepancies between the two primary statistical reports, these will be identified and we will then consider which is the most correct result. A final statistical report will be prepared, and all two (or three, if anything is to be corrected) statistical reports will be published as a supplementary material. Mock tables are presented in Supplementary material 1.

## Discussion

The primary aim of this paper is to minimise the risks of bias associated with selective outcome reporting and erroneous data-driven results. We therefore present a pre-defined statistical analysis plan for the MBT-RCT trial.

### Strengths

Our methodology has several strengths. First, our methodology is pre-defined, and our analyses will adhere to this statistical analysis plan. Second, we have limited problems with multiplicity because we only assess one primary outcome, and our conclusions will primarily be based on the results of the primary outcome [29]. Third, all analyses will be conducted according to the intention-to-treat principle, and, if necessary, we will use multiple imputations and best-worst/worst-best case scenarios to assess the potential impact of missing data [35]. Furthermore, we plan to systematically assess if the underlying statistical assumptions are fulfilled for all statistical analyses.

### Limitations

A potential limitation of the MBT-RCT trial is that no systematic review of the effects of short-term compared to long-term psychotherapy for borderline personality disorder, or for psychiatric disorders in general, was available prior to planning of this trial. Hence, estimations of anticipated intervention effects, estimations of variances used in our sample size, and power estimations, etc. may be erroneous. We are currently performing such a review, which will be submitted for publication prior to completion of this trial [40]. Second, we expect a significant amount of missing data, due to the instability of the trial population. Even though we plan to handle missing data appropriately, no statistical method can guarantee the validity of trial results if the missingness is substantial. Third, even though the trial will be sufficiently powered to confirm or reject intervention effects on the primary and secondary outcomes, the relatively small number of randomised participants may result in a risk of baseline differences which also may bias the trial results especially on non-primary outcomes. However, we will carefully consider the low sample size when interpreting the trial results. Fourth, as participants are not blinded to the allocated treatment, results from all participant-reported outcomes are at risk of bias [10]. Fifth, therapists are likewise not blinded to the allocated treatment and may have an allegiance to one of the interventions. Sixth, we have planned several subgroup analyses. However, subgroup analyses are per definition underpowered, and will be considered hypothesis-generating only. We will carefully consider these limitations when interpreting the results.

## Conclusion

We have developed this statistical analysis plan in line with the Declaration of Helsinki and the International Conference on Harmonization of Good Clinical Practice Guidelines, which should increase the validity of the MBT-RCT trial by mitigation of analysis bias.

## Supplementary Information

**Additional file 1: Table S1.** Baseline characteristics of the trial population. BPD; Borderline Personality Disorder; MINI: Mini International Neuropsychiatric Interview; No.: Number; SCID-5-PD: Structured Clinical Interview for DSM-5 Personality Disorders; SD: Standard Deviation. **Table S2.** Primary and secondary outcome results (ITT Population). GAF: Global Assessment of Functioning; ITT: Intention To Treat; SF-36: Short Form Health Survey – 36; SD: Standard Deviation; WSAS: Work and Social Adjustment Scale; ZAN-BPD: Zanarini Rating Scale for Borderline Personality Disorder.

## Data Availability

Trial investigators, the steering committee, and statisticians at the Copenhagen Trial Unit will have access to the data. After the end of the trial, approval for making the final dataset publicly available in a depersonalised format in the Danish Data Archive will be applied for through the Danish Data Protection Agency.
