# Supplementary material for: Detailed statistical analysis plan for the short-term versus long-term mentalisation-based therapy for outpatients with subthreshold or diagnosed borderline personality disorder randomised clinical trial (MBT-RCT)
Source: Trials. 2021 Jul 28;22:497. doi: 10.1186/s13063-021-05450-y (PMC8316699; doi:10.1186/s13063-021-05450-y)
Supplement: Supplementary file 1 — Additional file 1: Table S1. Baseline characteristics of the trial population. BPD; Borderline Personality Disorder; MINI: Mini International Neuropsychiatric Interview; No.: Number; SCID-5-PD: Structured Clinical Interview for DSM-5 Personality Disorders; SD: Standard Deviation. Table S2. Primary and secondary outcome results (ITT Population). GAF: Global Assessment of Functioning; ITT: Intention To Treat; SF-36: Short Form Health Survey – 36; SD: Standard Deviation; WSAS: Work and Social Adjustment Scale; ZAN-BPD: Zanarini Rating Scale for Borderline Personality Disorder. [file 13063_2021_5450_MOESM1_ESM.docx]

**Table 1**

Baseline characteristics of the trial population

| Characteristic | Short-term MBT  (n=83) | Long-term MBT (n=83) |
| --- | --- | --- |
| Demographic characteristics |  |  |
| Age, mean (SD), years | XX (X) | XX (X) |
| Female sex – no. (%) | XX (X) | XX (X) |
| Civil status – no. (%) |  |  |
| Married | X (X) | X (X) |
| Living together | X (X) | X (X) |
| Single | X (X) | X (X) |
| Living situation – no. (%) |  |  |
| Alone | X (X) | X (X) |
| With others | X (X) | X (X) |
| Education level after high school – no. (%) |  |  |
| Currently studying | X (X) | X (X) |
| Short education | X (X) | X (X) |
| Medium education | X (X) | X (X) |
| Long education | X (X) | X (X) |
| No higher education | X (X) | X (X) |
| Employment status – no. (%) |  |  |
| On social welfare | X (X) | X (X) |
| Part time employed | X (X) | X (X) |
| Employed | X (X) | X (X) |
| Unemployed/homemaker | X (X) | X (X) |
| Clinical characteristics |  |  |
| Psychiatric comorbidity (MINI) – no. (%) |  |  |
| Anxiety disorders | X (X) | X (X) |
| Major depressive disorder | X (X) | X (X) |
| Post-traumatic stress disorder | X (X) | X (X) |
| Proportion of participants with subthreshold BPD – no. (%) | X (X) | X (X) |
| No. of BPD criteria (SCID-5-PD), mean (SD) | X (X) | X (X) |
| Personality disorder comorbidity (SCID-5-PD) – no. (%) |  |  |
| Avoidant personality disorder | X (X) | X (X) |
| Obsessive-compulsive personality disorder | X (X) | X (X) |
| Dependent personality disorder | X (X) | X (X) |
| Paranoid personality disorder | X (X) | X (X) |
| Proportion of participants with one or more suicide-attempts the past 8 months – no. (%) | X (X) | X (X) |
| Proportion of participants with one or more acts of severe self-harm the past 8 months – no. (%) | X (X) | X (X) |
| Proportion of participants on antidepressants | X (X) | X (X) |
| Proportion of participants on antipsychotics |  | X (X) |
| Proportion of participants on other psychoactive drugs | X (X) | X (X) |
| Mean (SD) number of days from randomization to assessment time-point |  |  |
| 8 months post-randomization | X (X) | X (X) |
| 16 months post-randomization | X (X) | X (X) |
| 24 months post-randomization | X (X) | X (X) |

BPD; Borderline Personality Disorder; MINI: Mini International Neuropsychiatric Interview; No.: Number; SCID-5-PD: Structured Clinical Interview for DSM-5 Personality Disorders; SD: Standard Deviation

**Table 2**

Primary and secondary outcome results (ITT Population)

| **Outcome** | Short-term MBT group  (n= 83) | | Long-term MBT group  (n= 83) | | Estimate (95% CI) | *p*-value |
| --- | --- | --- | --- | --- | --- | --- |
|  | No. analyzed | Result | No. analyzed | Result |  |  |
| **Primary** |  |  |  |  |  |  |
| Borderline symptoms, ZAN-BPD, mean (SD) |  |  |  |  |  |  |
| - Baseline | X | XX (X) | X | XX (X) | Mean difference (XX to YY) | *p = ZZ* |
| - 8 months | X | XX (X) | X | XX (X) | Mean difference (XX to YY) | *p = ZZ* |
| - 16 months | X | XX (X) | X | XX (X) | Mean difference (XX to YY) | *p = ZZ* |
| **Secondary** |  |  |  |  |  |  |
| Functional impairment, WSAS, mean (SD) |  |  |  |  |  |  |
| - Baseline | X | XX (X) | X | XX (X) | Mean difference (XX to YY) | *p = ZZ* |
| - 8 months | X | XX (X) | X | XX (X) | Mean difference (XX to YY) | *p = ZZ* |
| - 16 months | X | XX (X) | X | XX (X) | Mean difference (XX to YY) | *p = ZZ* |
| Quality of life, SF-36 – mental component, mean (SD) |  |  |  |  |  |  |
| - Baseline | X | XX (X) | X | XX (X) | Mean difference (XX to YY) | *p = ZZ* |
| - 8 months | X | XX (X) | X | XX (X) | Mean difference (XX to YY) | *p = ZZ* |
| - 16 months | X | XX (X) | X | XX (X) | Mean difference (XX to YY) | *p = ZZ* |
| Global functioning, GAF, mean (SD) |  |  |  |  |  |  |
| - Baseline | X | XX (X) | X | XX (X) | Mean difference (XX to YY) | *p = ZZ* |
| - 8 months | X | XX (X) | X | XX (X) | Mean difference (XX to YY) | *p = ZZ* |
| - 16 months | X | XX (X) | X | XX (X) | Mean difference (XX to YY) | *p = ZZ* |
| Proportion of participants with severe self-harm the past 8 months |  |  |  |  |  |  |
| - Baseline | X | XX/YY (ZZ%) | x | XX/YY (ZZ%) | Relative risk XX (YY to ZZ) | *p = ZZ* |
| - 8 months | X | XX/YY (ZZ%) | x | XX/YY (ZZ%) | Relative risk XX (YY to ZZ) | *p = ZZ* |
| - 16 months | X | XX/YY (ZZ%) | x | XX/YY (ZZ%) | Relative risk XX (YY to ZZ) | *p = ZZ* |

GAF: Global Assessment of Functioning; ITT: Intention To Treat; SF-36: Short Form Health Survey – 36; SD: Standard Deviation; WSAS: Work and Social Adjustment Scale; ZAN-BPD: Zanarini Rating Scale for Borderline Personality Disorder
